# Feasibility and Efficacy of Virtual Reality Interventions to Improve Psychosocial Functioning in Psychosis: Systematic Review

**DOI:** 10.2196/28502

**Published:** 2022-02-18

**Authors:** Alexandra H Schroeder, Bryce J M Bogie, Tabassum T Rahman, Alexandra Thérond, Hannah Matheson, Synthia Guimond

**Affiliations:** 1 The Royal’s Institute of Mental Health Research University of Ottawa Ottawa, ON Canada; 2 Department of Neuroscience Carleton University Ottawa, ON Canada; 3 Department of Cellular and Molecular Medicine, Faculty of Medicine University of Ottawa Ottawa, ON Canada; 4 Department of Psychology Université du Québec à Montréal Montréal, QC Canada; 5 Department of Psychology Carleton University Ottawa, ON Canada; 6 Department of Psychoeducation and Psychology University of Quebec in Outaouais Gatineau, QC Canada; 7 Department of Psychiatry University of Ottawa Ottawa, ON Canada

**Keywords:** auditory verbal hallucinations, cognitive remediation, functional outcomes, neurocognition, paranoia, psychosis, schizophrenia, social skills, virtual reality (VR), vocational skills

## Abstract

**Background:**

Functional recovery in psychosis remains a challenge despite current evidence-based treatment approaches. To address this problem, innovative interventions using virtual reality (VR) have recently been developed. VR technologies have enabled the development of realistic environments in which individuals with psychosis can receive psychosocial treatment interventions in more ecological settings than traditional clinics. These interventions may therefore increase the transfer of learned psychosocial skills to real-world environments, thereby promoting long-term functional recovery. However, the overall feasibility and efficacy of such interventions within the psychosis population remain unclear.

**Objective:**

This systematic review aims to investigate whether VR-based psychosocial interventions are feasible and enjoyable for individuals with psychosis, synthesize current evidence on the efficacy of VR-based psychosocial interventions for psychosis, and identify the limitations in the current literature to guide future research.

**Methods:**

This research followed the PRISMA (Preferred Reporting Items for Systematic Reviews and Meta-Analyses) guidelines. Literature searches were conducted in PubMed and PsycINFO in May 2021. We searched for peer-reviewed English articles that used a psychosocial intervention with a VR component. Participants in the included studies were diagnosed with schizophrenia, schizoaffective disorder, or another psychotic disorder. The included studies were divided into four categories as follows: cognitive remediation interventions, social skills interventions, vocational skills interventions, and auditory verbal hallucinations and paranoia interventions. The risk of bias assessment was performed for each study.

**Results:**

A total of 18 studies were included in this systematic review. Of these 18 studies, 4 (22%) studies used a cognitive remediation intervention, 4 (22%) studies used a social skills intervention, 3 (17%) studies used a vocational skills intervention, and 7 (39%) studies implemented an intervention aimed at improving auditory verbal hallucinations or paranoia. A total of 745 individuals with psychosis were included in the study. All the studies that evaluated feasibility showed that VR-based psychosocial interventions were feasible and enjoyable for individuals with psychosis. The preliminary evidence on efficacy included in this review suggests that VR-based psychosocial interventions can improve cognitive, social, and vocational skills in individuals with psychosis. VR-based interventions may also improve the symptoms of auditory verbal hallucinations and paranoia. The skills that participants learned through these interventions were durable, transferred into real-world environments, and led to improved functional outcomes, such as autonomy, managing housework, and work performance.

**Conclusions:**

VR-based interventions may represent a novel and efficacious approach for improving psychosocial functioning in psychosis. Therefore, VR-based psychosocial interventions represent a promising adjunctive therapy for the treatment of psychosis, which may be used to improve psychosocial skills, community functioning, and quality of life.

## Introduction

### Background

Psychosis is a mental state characterized by hallucinations, delusions, disorganized thoughts, disorganized speech, and disorganized or catatonic behavior [1]. People with psychosis often experience social and cognitive impairments [2,3]. This panoply of symptoms is associated with widespread debilitating effects on functioning in people with psychosis [4-6]. Although current pharmacological treatments (ie, antipsychotic medications) are often successful in remediating the positive symptoms related to psychosis, some individuals experience persistent symptoms, including auditory verbal hallucinations and paranoia [7,8]. Furthermore, antipsychotic medications have shown limited efficacy in improving cognitive and psychosocial functioning [9-12]. Hence, adjunctive interventions aimed at improving psychosocial outcomes are critical for the comprehensive and holistic treatment of psychosis [13,14].

Existing psychosocial interventions used in patients with psychosis typically focus on improving cognitive, social, or vocational skills [15,16]. Cognitive interventions target improvements in various cognitive domains, including attention, executive function, and working and verbal memory [17,18]. Social skills interventions target social cognition and social skills through technology, cognitive behavioral techniques, psychoeducation, and life management skills [15]. Finally, vocational skills interventions specifically aim to improve employment rates for individuals with psychosis [16,19]. These interventions have been shown to improve symptomatic *and* functional outcomes in individuals with psychosis [15,16]. However, the long-term maintenance of these positive outcomes, along with the generalizability and transfer of learned skills to real-world environments, remains a challenge [14,15,20]. For instance, current psychosocial interventions for individuals with psychosis are often offered in clinical settings, which may restrict the transfer of learned skills outside of the clinic [21,22]. Therefore, the development of more ecological interventions is needed.

Over the past decade, advancements in virtual reality (VR) technology have expanded the types of psychosocial interventions that can be offered to patients with psychosis [23,24]. VR involves computer technology that enables the perception of multisensory stimuli within immersive, 3D, complex environments [25,26]. With VR, patients can practice functioning in familiar settings, which may allow them to develop skills that are more generalizable to real-world situations [27,28]. Furthermore, health care providers can interact with patients in the VR environment in real time, offering in situ therapy, support, and guidance [27]. These features of VR technology have previously been used in the treatment of several psychiatric and medical conditions, including anxiety disorders (where VR has been integrated with cognitive behavioral therapy and exposure therapy) [29], traumatic brain injury [30], multiple sclerosis [31], and stroke [32]. More recently, VR-based interventions have been implemented to improve auditory verbal hallucinations and paranoia in psychosis, where traditional treatment approaches for these symptoms are limited to cognitive behavioral therapy, electroconvulsive therapy, or transcranial magnetic stimulation [33,34]. Hence, VR represents a promising tool for psychosocial interventions in psychosis because of its increased capacity for ecological validity and its potential to be more engaging than traditional interventions [24,32,35,36].

Although there is some empirical evidence suggesting that VR-based psychosocial interventions may improve symptoms and community functioning in individuals with psychosis [37,38], there are no systematic reviews focusing on this topic. Furthermore, there are known possible risks and side effects associated with the use of VR, including fatigue and simulator sickness [36]. However, the adverse effects of VR-based psychosocial interventions on psychosis have not been systematically investigated. Therefore, to fill these knowledge gaps, this study systematically reviewed the literature to explore the effects of VR-based psychosocial interventions in individuals with psychosis.

### Objectives

This systematic review aims to (1) investigate whether VR-based psychosocial interventions are feasible and enjoyable for individuals with psychosis, (2) synthesize current evidence on the efficacy of VR-based psychosocial interventions for psychosis, and (3) identify the limitations in the current literature to guide future research.

## Methods

This systematic review followed the PRISMA (Preferred Reporting Items for Systematic Reviews and Meta-Analyses) guidelines [39].

### Search Strategy

We conducted a broad search of the published literature using the following search strategy: (*treatment* OR *therapy* OR *intervention* OR *training* OR *rehabilitation*) AND (*schizophrenia* OR schizoaffective OR *psychosis*) AND (*virtual reality*). Searches were executed in PubMed and PsycINFO on May 20, 2021. Searches were limited to English language sources with no limits on setting, date, age group, or geographical restrictions. The reference lists of the included studies were reviewed to identify additional potential records.

### Eligibility Criteria

Studies were included if they (1) were a randomized controlled trial (RCT), randomized comparative trial, randomized partial crossover trial, nonrandomized controlled trial, or single-arm study; (2) used a psychosocial intervention with a VR component; and (3) included participants with schizophrenia, schizoaffective disorder, or other psychotic disorders. The inclusion of a control group was not an eligibility criterion. Studies were excluded if they (1) were not available in English; (2) were a review article, commentary, study protocol, case report, or conference paper; (3) included a mixed clinical population (eg, combined participants with a psychotic disorder and participants with a mood disorder); or (4) did not assess and compare pre- versus postintervention outcomes.

### Study Selection

**Figure 1:**
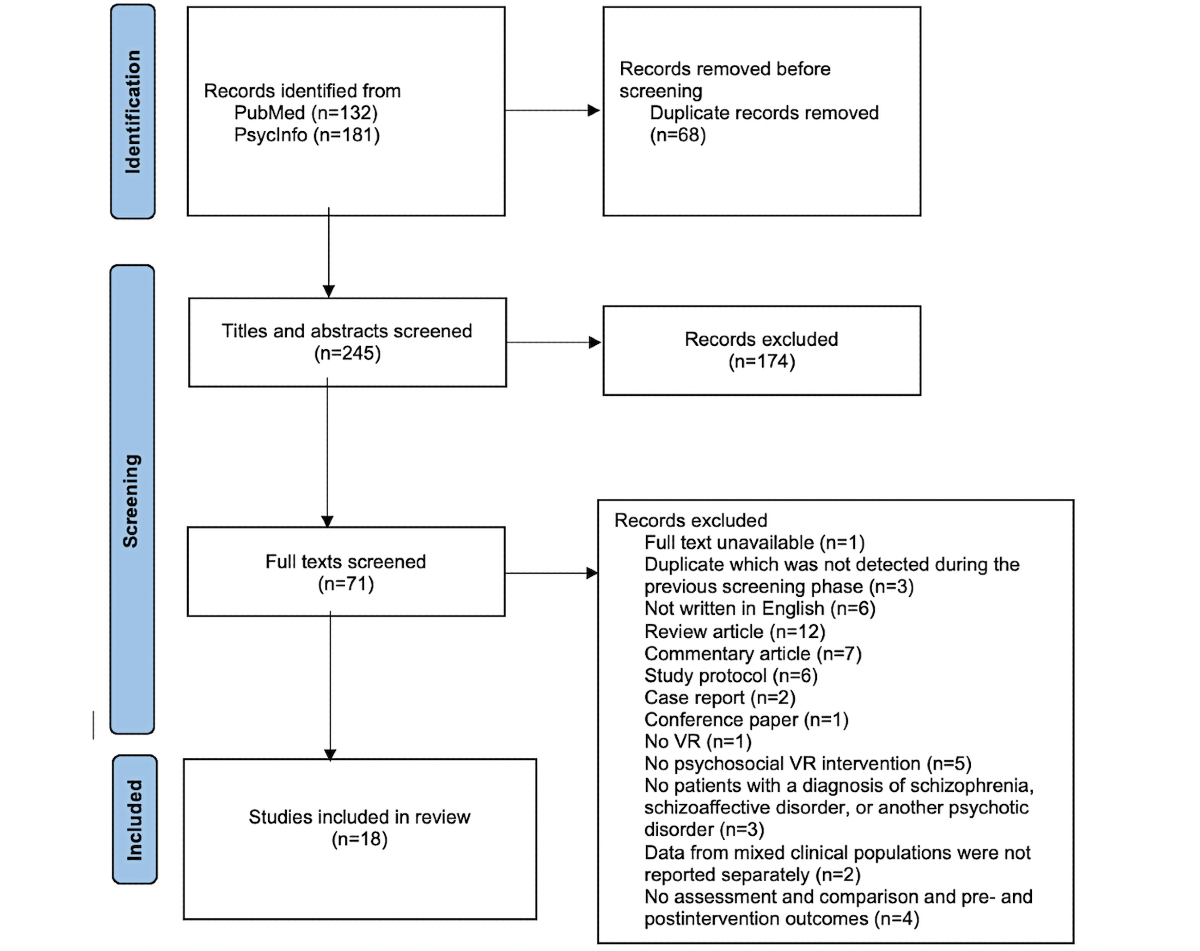
PRISMA (Preferred Reporting Items for Systematic Reviews and Meta-Analyses) flow diagram. VR: virtual reality.

Figure 1 summarizes the study selection process. We identified 313 records from our literature search (PubMed=132; PsycINFO=181). Once duplicates were removed, at least 2 authors (AHS, BJMB, TTR, AT, or HM) screened the titles and abstracts of the remaining 245 (78.3%) studies (disagreements were resolved through discussion until consensus was reached). A total of 71 (29%) studies met the eligibility criteria and subsequently underwent full-text review. At least two authors (AHS, BJMB, TTR, AT, or HM) screened each study (disagreements were resolved through discussion until consensus was reached). A total of 18 (25%) studies were included in the final narrative synthesis of this systematic review.

### Data Extraction

The following information was extracted from the included studies: author, year of publication, country, sample size, diagnosis of participants, mean age of participants, study design, blinding protocol, VR condition, control condition, number and duration of VR sessions, type of VR system, type of VR-based psychosocial interventions and exercises, feasibility, measured outcomes, and main findings. The extracted information was independently cross-checked by at least 2 authors (AHS, BJMB, TTR, AT, or HM).

### Risk of Bias Assessment

Risk of bias assessments for the included RCTs and the single randomized comparative trial was performed using the Cochrane risk-of-bias tool for randomized trials (version 2) [40]. Modified versions of the Cochrane risk-of-bias tool for randomized trials (version 2) were used to assess the risk of bias in a single randomized partial crossover trial [41] and single-arm studies [42]. Finally, the Newcastle-Ottawa Quality Assessment Scale was used to assess the risk of bias in nonrandomized controlled trials [43]. The risk of bias assessment for each study was independently evaluated by 2 authors (AHS, BJMB, AT, or HM). Disagreements were resolved through discussion until a consensus was reached. Multimedia Appendix 1 [40,42-61] provides more information on each tool used to assess the risk of bias in the included studies.

## Results

### General Characteristics of the Included Studies

Of the 18 studies included in this systematic review, 4 (22%) studies used a cognitive remediation intervention, 4 (22%) studies used a social skills intervention, 3 (17%) studies used a vocational skills intervention, and 7 (39%) studies used an intervention aimed at improving auditory verbal hallucinations or paranoia. Table 1 provides a general summary of the included studies. Table S1 in Multimedia Appendix 2 [44-61] provides further details of the studies included in this systematic review.

**Figure 2:**
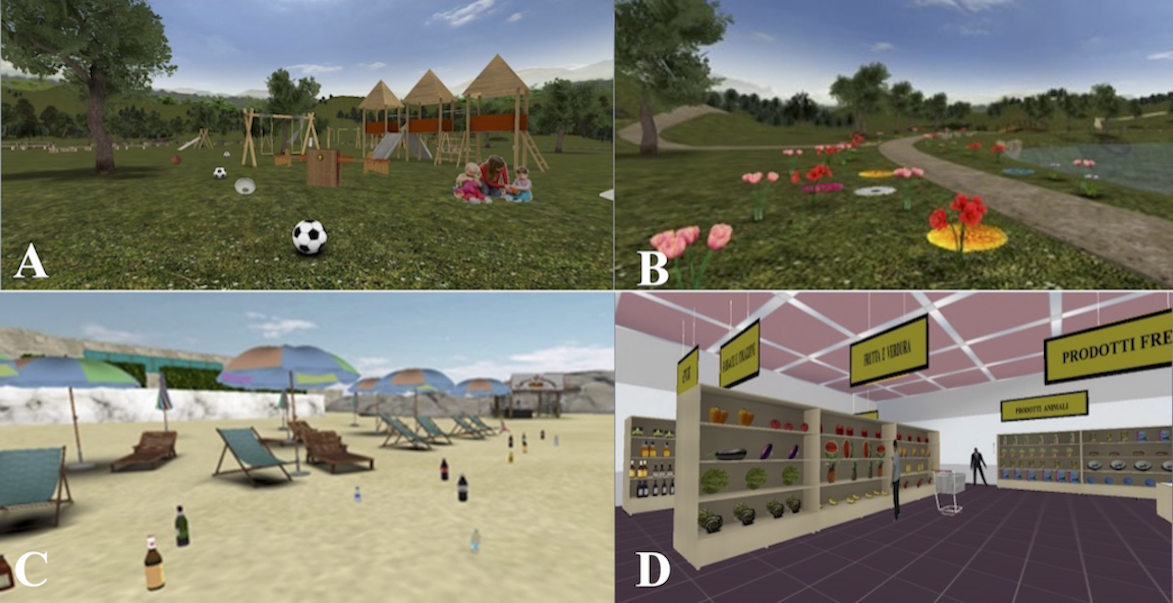
Virtual reality environments developed via the NeuroVr 2.0 software for a cognitive remediation intervention in schizophrenia. The environments include a park (A), valley (B), beach (C), and supermarket (D). Images reproduced with author permission from La Paglia [46].

A total of 3 of the 4 (75%) cognitive remediation studies required participants to complete daily life tasks in VR, which involved the use of common cognitive skills, such as shopping using a list or navigating in public transit [44,46,47]. The other cognitive remediation intervention used VR games to train the fluid intelligence [45]. Figure 2 provides an example of a VR environment used in a cognitive remediation intervention [47].

**Figure 3:**
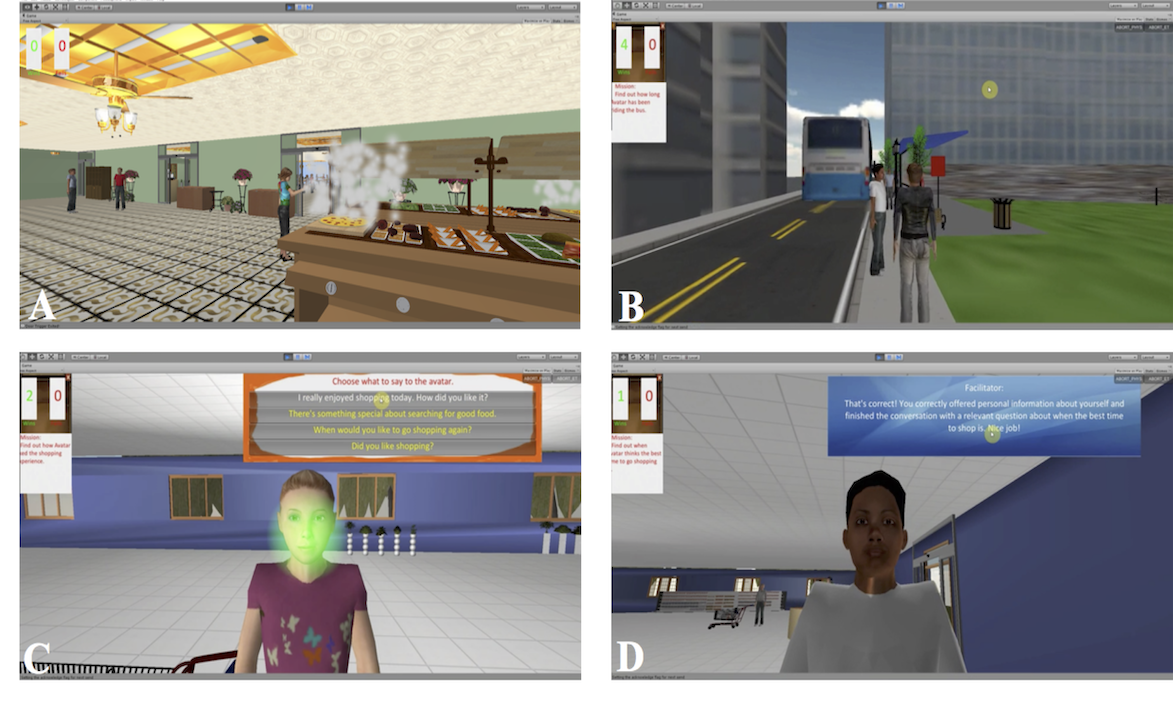
Virtual reality environment used in the Multimodal Adaptive Social Intervention in virtual reality. (A) The cafeteria environment and (B) the bus stop environment where participants can practice "small talk" and interact with the avatars. (C) The participant chooses what to say to the avatar from a multiple-choice menu. (D) The avatar provides feedback after the exchange with the participant. Reprinted from Psychiatry Research, Adery et al [48], Copyright 2018, with permission from Elsevier.

**Figure 4:**
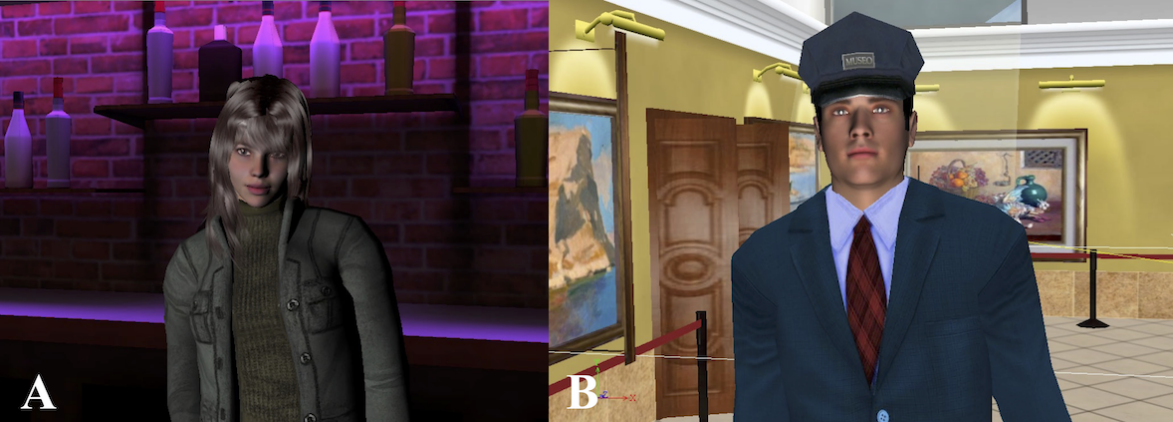
Virtual reality environment used in the Soskitrain virtual reality integrated program for improving social skills in patients with schizophrenia. Participants can practice social interactions with avatars, such as (A) a bartender and (B) a security guard in a museum. Unpublished images reproduced from the Soskitrain program with author permission from Rus-Calafell [50].

All social skills interventions required participants to interact socially with the avatars. Feedback was provided to the participants by the avatars or the therapist during the VR intervention [48-51]. Figures 3 and 4 provide examples of VR environments used in social skills interventions [48,50].

In all, 2 (67%) of the vocational skills intervention studies focused on developing work-related skills in specific job environments, such as in a boutique [54], convenience store, and supermarket [53]. The other vocational skills intervention study trained participants on job interviewing skills [52]. The VR scenarios were chosen based on common settings in which individuals with mental illness might find employment [53,54].

Within the auditory verbal hallucinations or paranoia category, 3 studies used the same intervention in which participants created avatars that most accurately resembled the entity they believed was the source of their hallucinations [55-57]. Participants then engaged in a dialogue with these avatars, which were animated in real time by a therapist. Within this category, 3 other studies also used the same intervention that involved VR-based cognitive behavioral therapy to reduce paranoia and improve social participation [58,60,61]. In these studies, participants interacted with avatars in VR-based social environments while simultaneously communicating with a therapist. Finally, the remaining study used an intervention in which participants met pedestrians while navigating through a virtual street [59]. Participants subsequently had to recollect the pedestrians’ facial affect. Participants graded their confidence in their responses and received feedback on the accuracy of their memory performance. The feedback served to induce doubt in the participants about their false judgments or errors from the memory task, which was hypothesized to affect the participants’ overall judgment process. The aim of this study was to assess the effect of this intervention on delusion severity (ie, the severity of false, fixed beliefs that are resistant to counterevidence).

**Table 1:** Summary of included studies.

Study (country)		Sample	Diagnosis of participants	Study design	Control condition	VR^a^ exercises	Main findings
**Cognitive remediation interventions**							
	Amado et al [44] (France)	N^b^=7, *k*^c^=1	Schizophrenia or schizoaffective disorder	Single-arm, unblinded, pilot study	N/A^d^	Participants acted as pedestrians in a virtual town and completed tasks that were difficult for them in real life (eg, memorizing an itinerary, shopping, or being on time at a meeting point). After the exercise, participants discussed the possible transfer of skills to their real lives and were assigned a task to perform at home.	Pre- and postintervention assessments: the VR group showed improvements in attention, working memory, prospective memory, retrospective memory, and autonomy. There were no improvements in planning. Postintervention qualitative findings: the VR group reported improvements in their amount of energy to develop concrete plans to look for employment or to return to community activities, sparing time, planning, enriched relatedness, and management of their housework.
	Chan et al [45] (Hong Kong)	N=27*, k*=2; experimental: n^e^=12; control: n=15	Schizophrenia	Randomized controlled trial, pilot study	Treatment as usual (attended the usual program in the long-term care facility; did not include VR)	The (1) ball and bird and (2) shark bait activities were chosen to train fluid intelligence. Ball and bird activity: balls of different colors fly toward participants, and they must contact the ball using any part of their body, making the ball “burst” or “transform” into doves. Shark bait activity: participants navigate in the sea and chase a yellow star while avoiding distracters.	Compared with the control group, the VR group showed significant improvements in overall cognitive function, repetition, and memory.
	La Paglia et al [46] (Italy)	N=12, *k*=2; experimental: n=6; control: n=6	Schizophrenia	Nonrandomized controlled trial, pilot study	IPT^f^ and pharmacological therapy	Tasks to train attention and executive function in VR environments such as a park, valley, beach, and supermarket. For example, participants collected and bought products from a shopping list in a supermarket setting to train executive function.	Pre- and postintervention assessments: both groups showed significant benefits in divided attention. The VR group also showed reduced cognitive deficits and improved planning. After the executive function training (VR supermarket), the experimental group showed improvements in decreased errors, reduced time of execution, and increased observance of rules. After the attention training (VR park, valley, and beach), the experimental group showed improvements in reduced time of execution, decreased perseverative errors, and improved sustained attention.
	La Paglia et al [47] (Italy)	N=15, *k*=2; experimental: n=9; control: n=6	Schizophrenia	Nonrandomized controlled trial, pilot study	IPT and pharmacological therapy	Three different virtual environments (park, valley, and beach) featured hierarchical sequences of tasks designed to train attention (eg, catching footballs that were presented at irregular time intervals, identifying and picking a specific type of flower, or picking up specific types of bottles while being alerted to calls and loudspeaker announcements).	Pre- and postintervention assessments: both groups showed improvements in divided attention. The VR group showed improvements in general cognitive functioning, planning, sustained attention, reduced time of execution, decreased requests for assistance, decreased needs of the therapist’s intervention, and decreased number of omissions.
**Social skills interventions**							
	Adery et al [48] (United States)	N=16, *k*=1	Schizophrenia	Single-arm, single blind, feasibility study	N/A	Three different VR environments were used (bus stop, shop, and cafeteria) to train participants in both microlevel social skills (ie, eye contact and facial expression) and macrolevel social skills (ie, starting conversations and requesting help). Tasks were administered without time constraints.	Pre- and postintervention assessments: the VR group showed improvements in overall clinical symptoms and negative symptoms.
	Park et al [49] (Republic of Korea)	N=64, *k*=2; SST-VR^g^: n=33; SST-TR^h^: n=31	Schizophrenia	Randomized controlled trial, single blind, efficacy study	Social skills training using traditional role-playing (in-person with the social skills training therapist as the role-play actor instead of using VR)	Role-play with virtual avatars in environments such as a restaurant or walking down a street. Participants were trained in conversation skills, assertive skills, and emotional expression skills. Helper avatars provided positive and corrective feedback as needed.	Compared with the control group, the VR group showed improvements in conversational skills and assertiveness. Compared with the VR group, the control group showed improvements in nonverbal skills.
	Rus-Calafell et al [50] (Spain)	N=12, *k*=1	Schizophrenia or schizoaffective disorder with a deficit in social skills or social functioning	Single-arm, unblinded, pilot study	N/A	The VR program comprised 7 activities that each targeted different social skills. Participants received positive or negative reinforcement from virtual avatars based on their performance in a bar or supermarket environment.	Pre- and postintervention assessments: the VR group showed improvements in negative symptoms, psychopathology, social anxiety and discomfort, avoidance, social functioning, learning in emotion perception, assertive behaviors, and time spent in a conversation.
	Vass et al [51] (Hungary)	N=17, *k*=2; VR-ToMIS^i^: n=9; control: n=8	Schizophrenia	Randomized controlled pilot study	Passive VR control condition (participants used the same VR software as the experimental group but without any intervention)	The VR-based targeted theory of mind (ToM) intervention (VR-ToMIS) used cognitive and behavioral therapeutic techniques. Participants engaged in social interactions with an avatar with prerecorded dialogue that was designed to induce ToM impairment (double meaning sentences, overstatements, and irony). After the interaction, participants visualized the inferred emotions of the avatar. The task was also discussed with a therapist.	Pre- and postintervention assessments: VR-ToMIS was associated with improvements in negative symptoms, in 1 neurocognitive field (immediate memory), ToM, and pragmatic language skills, but no significant change in quality of life was detected. These findings were also significantly greater in the VR-ToMIS group compared with the control group.
**Vocational skills interventions**							
	Smith et al [52] (United States)	N=32, *k*=2; VR-JIT^j^: n=21; control: n=11	Schizophrenia or schizoaffective disorder	Randomized controlled trial, single blind, efficacy study	Treatment as usual (which did not include a VR component)	The VR-JIT was designed to improve interviewing skills. Participants completed virtual job interview role plays with a virtual human resources representative that were each approximately 20 minutes in duration.	Pre- and postintervention assessments: the VR group showed improvements in role-play job interview scores. Compared with the control group, the VR group showed improvements in the odds of receiving a job offer at a 6-month follow-up. There was also an association between more training and waiting fewer weeks to receive a job offer.
	Sohn et al [53] (Republic of Korea)	N=9, *k*=1	Schizophrenia	Single-arm, feasibility study	N/A	The virtual reality-based vocational rehabilitation training program included scenarios with a convenience store employee and supermarket clerk. Each scenario included various situations that trained participants on how to manage problems they may encounter in real life. For example, the convenience store situations included training on the arrangement of goods and training for problematic situations.	Pre- and postintervention assessments: the VR group showed improvements in individual and social performance, general symptoms, verbal memory, and immediate and delayed recall on visual memory. Improvements in positive symptoms showed a trend toward significance.
	Tsang and Man [54] (Hong Kong)	N=75, *k*=3; experimental: n=25; TAG^k^: n=25; CG^l^: n=25	Schizophrenia	Randomized controlled trial, single blind, efficacy study	TAG (received therapist-administered vocational training) and conventional treatment group (CG). Neither control group experienced a VR intervention.	The 3D nonimmersive VR training was set in a boutique. The training involved a hierarchical structure divided into levels in which problem-solving competence tests had to be passed to advance levels (pretrainee level, trainee level, and sales level).	Compared with both control groups, the VR group showed improvements in cognitive functioning and executive functioning performance. Compared with the CG, the VR group showed improvements in self-efficacy. Compared with the CG, the VR and TAG groups showed improvements in work performance during an on-site test.
**Auditory verbal hallucinations and paranoia interventions**							
	Dellazizzo et al [55] (Canada)	N=10, *k*=1	Schizophrenia or schizoaffective disorder	Single-arm study	N/A	Participants who had already undergone CBT^m^ as part of the study by Dellazizzo et al [56] were invited to complete the VR intervention (ie, after finishing CBT). The VR intervention was identical to that used in Dellazizzo et al [56], which is described further in the table.	The VR group showed improvements in auditory verbal hallucinations, beliefs about voices, depressive symptoms, symptoms of schizophrenia, and quality of life.
	Dellazizzo et al [56] (Canada)	N=74, *k*=2; experimental: n=37; control: n=37	Schizophrenia or schizoaffective disorder	Randomized comparative trial, pilot study	CBT with no VR component	Participants created avatars which they believed most resembled the entity which was the source of their most distressing or dominant auditory verbal hallucination. Participants were encouraged to enter a dialogue with their avatar (which was animated in real time by a therapist). The interaction with the avatar became more supportive and less abusive as the intervention progressed. The conversations were designed to target participants’ emotional regulation, assertiveness, and self-esteem.	Both groups showed improvements in the severity of their auditory verbal hallucinations and depressive symptoms. The VR group also showed improvements in persecutory beliefs and quality of life. Although the results did not show a statistically significant superiority of the VR intervention over CBT in improving auditory verbal hallucinations, the VR intervention did achieve larger effect sizes, particularly on improving overall auditory verbal hallucinations. The VR intervention was superior to CBT at improving affective symptoms.
	du Sert et al [57] (Canada)	N=15, *k*=1	Schizophrenia or schizoaffective disorder	Randomized, partial crossover trial, pilot study	Treatment as usual (antipsychotic treatment and meetings with clinicians; no VR component).^n^	Participants created avatars which they believed most resembled the entity which was the source of their most distressing or dominant auditory verbal hallucination. Participants were encouraged to enter a dialogue with their avatar (which was animated in real time by a therapist). The interaction with the avatar became more supportive and less abusive as the intervention progressed. The conversations were designed to target participants’ emotional regulation, assertiveness, and self-esteem. ^n^	Pre- and postintervention assessments: the VR group showed improvements in auditory verbal hallucinations, beliefs about voices, general symptoms (however, positive and negative symptoms did not significantly improve), and quality of life.
	Geraets et al [58] (Netherlands)	N=91, *k*=2; experimental: n=43; control: n=48	Schizophrenia, schizoaffective disorder, or not-otherwise specified psychotic disorder	Randomized controlled trial, single blind	Treatment as usual with no VR component	VR-based CBT for reducing paranoia and improving social participation was used in this study. Evidence-based CBT elements were used by trained psychologists and exercises and behavioral experiments were completed in VR. Participants interacted with human avatars in social environments (a street, bus, café, and supermarket). Characteristics of the social environments (number of avatars and avatars’ responses to the participant) could be edited by the therapist, and they communicated with the participant during the VR sessions.	Pre- and postintervention assessments (baseline vs 3-month follow-up): compared with the control group, the VR group showed improvements in average levels of paranoia (feeling suspicious, disliked, and hurt) and negative affect (feeling anxious). Pre- and postintervention assessments (baseline vs 6-month follow-up): compared with the control group, the VR group showed improvements in average levels of paranoia (feeling disliked and hurt) and negative affect (feeling down and insecure). Positive affect did not improve more in the VR group than in the control group. The VR intervention did not change the interplay between affective states and paranoia.
	Moritz et al [59] (Germany)	N=33, *k*=1	Schizophrenia	Single-arm, proof of concept study	N/A	Participants met 6 different pedestrians while navigating through a virtual street on 2 occasions (in addition to 1 practice trial) in either a noise or no noise condition. Then, participants participated in a recognition task graded for confidence where they were asked to recollect the pedestrians and their corresponding facial affect. Participants also received feedback on the accuracy of their recall.	Pre- and postintervention assessments: the VR group showed improvements in paranoia. Improvement was associated with lower confidence ratings (both during the experiment, particularly for incorrect responses, and according to retrospective assessment).
	Pot-Kolder et al [60] (Netherlands)	N=116, *k*=2; experimental: n=58; control: n=58	Schizophrenia, schizoaffective disorder, delusional disorder, or not-otherwise specified psychotic disorder	Randomized controlled trial, single blind	Treatment as usual with no VR component	VR-based CBT for reducing paranoia and improving social participation was used in this study. Evidence-based CBT elements were used by trained psychologists and exercises and behavioral experiments were completed in VR. Participants interacted with human avatars in social environments (a street, bus, café, and supermarket). Characteristics of the social environments (number of avatars and avatars’ responses to the participant) could be edited by the therapist and, they communicated with the participant during the VR sessions.	Pre- and postintervention assessments (baseline vs 3-month follow-up): compared with the control group, the VR group showed improvements in momentary paranoid ideation when in the presence of others and momentary anxiety when in the presence of others. The VR group did not show a significant improvement in the amount of time spent with others. Pre- and postintervention assessments (baseline vs 6-month follow-up): the improvements shown at the 3-month follow-up were maintained at the 6-month follow-up. At the postintervention and follow-up time points, quality of life did not differ significantly among groups.
	Pot-Kolder et al [61] (Netherlands). ^o^	See Pot-Kolder et al [60]	See Pot-Kolder et al [60]	See Pot-Kolder et al [60]	See Pot-Kolder et al [60]	See Pot-Kolder et al [60] for the specific VR exercises used in this study. The main outcomes of this study related to the feasibility of the VR intervention.	Feasibility of the VR intervention: the average incremental cost per quality-adjusted life year was €48,868 (US $55,220.84). When relevant baseline differences were included, the average cost per quality-adjusted life year gained was €42,030 (US $47,493.9).

^a^VR: virtual reality.

^b^N: total sample size.

^c^k: number of groups.

^d^N/A: not applicable.

^e^n: group sample size.

^f^ITP: integrated psychological treatment.

^g^SST-VR: social skills training using VR role-playing.

^h^SST-TR: social skills training using traditional role-playing.

^i^VR-ToMIS: virtual reality-based targeted theory of mind intervention.

^j^VR-JIT: virtual reality job interview training.

^k^TAG: therapy administered group.

^l^CG: conventional group.

^m^CBT: cognitive behavioral therapy.

^n^The group comprised participants who received an “immediate” VR intervention as well as participants who received “delayed” VR intervention (that is, after they participated within a control, treatment as usual group). Both VR interventions were identical.

^o^This study used the same sample as Pot-Kolder et al [60]. This study reported novel outcomes.

### Feasibility

Overall, the VR-based psychosocial interventions included in this systematic review were associated with positive acceptability and feasibility profiles. For example, most participants in the VR-based cognitive remediation studies were largely satisfied with the interventions as shown through high attendance, punctuality, and participant questionnaire data [44,45]. Dropout rates ranged from 0% to 20% [44,45]. However, it should be noted that 2 of the 4 studies within this category did not specifically evaluate the feasibility of their intervention [46,47]. For the VR-based social skills interventions, participants reported positive feedback after using the VR system [48-51]. Dropout rates during VR-based social skills interventions ranged from 8% to 20% [48,50]. One study also showed higher attendance in the VR group than in the traditional social skills control group [49]. Most participants found VR-based vocational skills interventions helpful [52,54] and interesting [54]. One participant in a VR-based vocational skills intervention experienced simulator sickness, but this was not a problem throughout the rest of the training [54]. In the same study, some participants (particularly those who were less educated, chronically ill with long-term deinstitutionalization, and who rarely used computers) experienced different degrees of computer phobia [54]. Finally, in one of the studies that implemented an intervention aimed at improving auditory verbal hallucinations or paranoia, participants dropped out because they were too afraid of the intervention (n=1), found the head-mounted display too uncomfortable (n=2), or because they felt nauseous (n=1) [60]. The remaining studies that evaluated feasibility within this category reported positive feasibility profiles (Table S1 in Multimedia Appendix 2).

### Initial Evidence of Efficacy

The most commonly reported benefits following VR-based cognitive remediation interventions were improved memory [44,45], attention [44,46,47], and planning [44,46,47]. The single RCT and both nonrandomized controlled trials included within this category showed improvements in overall cognitive functioning in the VR group compared with the control group (treatment as usual, pharmacological therapy, and integrated psychological treatment) [45-47].

Improvements in conversational or communication skills [49,50], assertiveness [49,50], and negative symptoms [48,50,51] were observed following VR-based social skills interventions. In 1 RCT, the VR group showed more improvement in conversational skills and assertiveness but less improvement in nonverbal skills, compared with the active control group (social skills training using traditional role-playing) [49]. In the same RCT, the scores for generalization of skills were also higher in the VR group than in the active control group [49]. Another RCT, which targeted theory of mind (ToM), reported that the VR group displayed improved ToM, immediate memory, and pragmatic language skills compared with the passive VR control condition (wherein participants used the same VR system as the experimental group without the therapeutic intervention) [51].

One RCT showed that participants in the VR-based vocational skills intervention group displayed greater improvements in cognitive and executive functioning compared with both control groups (a therapist-administered training group and a conventional group) [54]. The other RCT showed that participants in the VR-based vocational skills intervention group had increased odds of receiving a job offer compared with the waitlist or treatment as usual control group [52]. The remaining study found that the VR-based intervention produced benefits in individual and social performance, general symptoms, verbal memory, and visual memory [53].

Finally, all VR-based psychosocial interventions for auditory verbal hallucinations or paranoia symptoms reported postintervention improvements. These improvements included reductions in the severity of auditory verbal hallucinations and persecutory beliefs [55,56], average levels of paranoia [58,59], momentary paranoid ideation [60], depressive symptoms [55,56], negative affect [58], and anxiety symptoms [60]. In the RCTs included within this category, the VR groups showed significant improvements compared with the control groups (treatment as usual) in the abovementioned outcomes [56,58,60,61]. However, in the randomized comparative trial included within this category [55], the VR-based intervention was not significantly better than cognitive behavioral therapy in improving the symptoms of auditory verbal hallucinations.

### Functional Outcomes

All types of VR-based interventions showed some efficacy in improving the functional outcomes. For instance, the VR-based cognitive skills intervention used by Amado et al [44] yielded postintervention benefits in several functional outcomes relating to autonomy. More specifically, qualitative participant reports revealed postintervention increases in energy with respect to developing plans, looking for employment, returning to community activities, and managing housework.

Studies by Park et al [49] and Rus-Calafell et al [50] showed improvements in functional outcomes following the VR-based social skills intervention, specifically on social functioning and conversation skills. However, the study by Vass et al [51] found no significant changes in quality of life following the VR-based targeted ToM intervention.

All 3 (100%) vocational rehabilitation studies showed improvements in functional outcomes [52-54], including benefits in work performance during an on-site test [54], role-play job interview scores [52], and social performance [53]. The study by Smith et al [52] conducted a 6-month follow-up to their intervention and observed that individuals who completed the job interview training in VR had increased odds of receiving a job offer compared with waitlist controls that received treatment as usual [52].

Finally, of the 5 studies that assessed quality of life in the auditory verbal hallucinations or paranoia studies category [55-57,60,61], 4 (80%) studies reported significant improvements in quality of life or quality-adjusted life years following the VR-based intervention [55-57,61]. The study by Pot-Kolder et al [60] also reported a significant improvement in social function in the VR group during a 6-month follow-up assessment.

### Durability

Of the included studies, 8 (44%) studies performed postintervention follow-up assessments or postintervention assessments in real-life environments to assess the durability of the interventions [49,50,52,54,56-58,60]. All 8 (100%) studies that assessed durability reported positive outcomes. For example, most of the skills that participants gained through the VR-based social skills interventions (eg, interpersonal communication skills and emotion perception skills) were maintained at a 4-month follow-up assessment [50]. In one of the RCTs included within the VR-based social skills intervention category, the generalization of skills was higher in the VR group than in the active control intervention (social skills training using traditional in-person role-playing with the therapist as the role-play actor instead of using VR) [49]. Similarly, at a 6-month follow-up, participants who received a VR-based vocational skills intervention had increased odds of receiving a job offer compared with controls who received treatment as usual [52]. The VR group also demonstrated stronger performance in sales-related activities (such as the ability to identify different items and the ability to sort clothes based on gender) compared with the control group during an on-site assessment [54]. Finally, participants who completed VR-based auditory verbal hallucinations or paranoia interventions showed reduced average levels of paranoia [58], negative affect [58], auditory verbal hallucinations [57], and severity of auditory verbal hallucinations [56] at follow-up time points (ranging from 3 to 12 months after the intervention).

### Overall Quality of the Included Studies

For the RCTs, the overall bias was low for 2 studies [52,54], some concerns were identified in 6 studies [45,49,51,58,60,61], and high concerns were identified in 1 study [56] (Table S1 in Multimedia Appendix 1). One nonrandomized controlled trial had a score of 5 out of 9 on the Newcastle-Ottawa Risk of Bias Scale [46], whereas the other had a score of 7 out of 9 [47] (Table S2 in Multimedia Appendix 1). In total, 5 single-arm studies had a low risk of bias [44,48,50,53,59], whereas 1 single-arm study had a high risk of bias [55] (Table S3 in Multimedia Appendix 1). Finally, there was a high risk of bias in the randomized partial crossover trial [57] (Table S4 in Multimedia Appendix 1). All studies identified as having a high risk of bias were included within the auditory verbal hallucinations or paranoia category.

## Discussion

### Principal Findings

The main finding of this systematic review was that VR-based interventions can be used as a feasible approach to improve psychosocial functioning in individuals with psychosis. All included studies showed significant improvements in at least one measured outcome after a VR-based psychosocial intervention was used. The RCTs included in this systematic review demonstrated significant improvements in overall cognitive function [45,54], conversational skills [49], and odds of receiving a job offer [52] following a VR-based intervention compared with a control condition. Within the included studies comparing a VR-based intervention to a traditional in-person rehabilitation condition, the VR interventions resulted in greater improvements in planning, cognitive function, sustained attention, conversational skills, assertiveness, and executive functioning (ie, the cognitive processes required for the cognitive control of goal-directed behavior) [47,49,54].

### Feasibility of VR-Based Psychosocial Interventions

Although VR technology has historically been an expensive and rare commodity, it is now affordable and can be administered in a cost-effective manner [61,62]. Our results suggest that VR is a safe and well-tolerated intervention that can be easily integrated into the treatment plan for individuals with psychosis. The studies included in this systematic review reported positive feasibility outcomes, such as high retention rates, participant satisfaction and motivation, and a low incidence of simulator sickness. Moreover, the study by Pot-Kolder et al [61] specifically examined the cost-effectiveness of VR-based cognitive behavioral therapy for psychosis and found that the intervention improved participant outcomes in a cost-effective manner. Therefore, the positive feasibility profiles of VR-based interventions support their integration into psychiatric clinics for individuals with psychosis.

### Impact of VR-Based Psychosocial Interventions for Individuals With Psychosis

The specific VR-based interventions evaluated within this systematic review included cognitive, social, and vocational skills interventions as well as interventions aimed at improving auditory verbal hallucinations or paranoia. We found that cognitive skills interventions were associated with improved memory [44,45], attention [44,46,47], planning [44,46,47], and overall cognitive functioning [45,47]. Findings from these studies suggest that individuals with psychosis can rehabilitate several cognitive domains in an ecologically valid environment through VR-based psychosocial interventions. Furthermore, we found that the most common benefits of VR-based social skills interventions were improvements in conversational skills [49,50], assertive behaviors [49,50], and negative symptoms [48,50,51]. Studies investigating VR-based vocational skills interventions varied in their methodology and trained either vocational skills or job interview skills. Nevertheless, all studies within this category have demonstrated significant improvements in vocational skills [52-54]. Finally, the interventions aimed at improving auditory verbal hallucinations or paranoia reported a reduction in the severity of auditory verbal hallucinations [55,56], average levels of paranoia [58,59], and momentary paranoid ideation [60].

Previous studies on rehabilitation interventions for individuals with psychosis have shown limitations in the generalizability and maintenance of skills in real-world environments [14,15,20]. Interestingly, in our current synthesis of the literature, we showed that generalizability, along with maintenance and transference of skills, to real-world environments is possible through the use of VR-based psychosocial interventions. Indeed, all 8 (100%) studies that assessed durability reported positive postintervention outcomes [49,50,52,54,56-58,60]. These findings demonstrate that the positive effects of VR-based interventions can be maintained months after the intervention has ended and that the skills gained during the intervention can be generalized to real-world environments. Given that cognitive, social, and vocational skills are strong predictors of quality of life, improving these skills in real-world environments may greatly enhance the lives of individuals with psychosis [3,20,63-65]. Further research on the generalizability and transference of skills to real-world environments must be conducted.

### Limitations

#### Limitations of This Systematic Review

This systematic review only assessed articles published in English, which limited the number of studies included. Furthermore, we only included peer-reviewed articles and did not examine any preprint servers for upcoming papers on the topics of interest.

#### Limitations of the Reviewed Literature

There are several limitations to the reviewed literature that must be noted. The number of identified studies within each VR-based intervention category was modest, and the sample sizes of the included studies were relatively small. There were also limitations in the number of studies that included active control conditions. This limited our ability to compare the efficacy of VR-based psychosocial interventions with that of their traditional counterparts. Furthermore, there was large heterogeneity across the VR environments (ie, immersive vs nonimmersive) and image quality used in the included studies. Immersiveness and image quality of the VR environment both influence user presence and emotional arousal, which may have affected the efficacy of the VR-based intervention as well as the transfer of skills to real-world settings [66,67]. There were also variations in the length and number of sessions used in each study, which could have affected the efficacy of the VR-based intervention. It is possible that longer VR sessions can result in participant fatigue and therefore reduce the efficacy of the intervention [68,69]. However, if the VR sessions are too short, the participant may not have sufficient time to become comfortable using the VR tool or to practice the desired skills, which may reduce the overall efficacy of the intervention [70]. Various methodological inconsistencies across the included studies prevented meta-analysis of the studies in this review.

### Recommendations for Future Research

On the basis of the methodological limitations identified in the current literature, future studies investigating the feasibility or efficacy of VR-based psychosocial interventions should aim to perform RCTs that compare a VR-based intervention to a traditional rehabilitation or active VR control condition. This would allow for direct comparisons of the efficacy of VR-based interventions to traditional rehabilitation interventions with respect to improvements in cognitive, social, and vocational skills as well as symptoms such as auditory verbal hallucinations and paranoia. Furthermore, the comparison of a VR experimental condition to a VR control condition would enable researchers to isolate the impact of the intervention in VR. Methodological consistency across future RCTs would also facilitate meta-analysis of the current evidence.

Future research should also include a varied duration and number of VR sessions to help determine the optimal number and duration of VR sessions to impact rehabilitation outcomes [71]. Finally, future research should include follow-up assessments to ensure that the skills gained during the intervention are maintained and transferred into the real world, especially, because the generalizability and maintenance of skills remain a concern for traditional rehabilitation interventions for individuals with psychosis [14,15,20].

A summary of the limitations of the reviewed literature and our recommendations for future research is shown in Table 2.

**Table 2:** Summary of the limitations in the current literature on virtual reality (VR)–based psychosocial interventions for individuals with psychosis and recommendations for future research.

Limitations in the current literature	Recommendations for future research	Impact of recommendation for future research
Limited number of studies comparing VR-based psychosocial interventions to traditional psychosocial interventions.	Perform RCTs^a^ that compare a VR-based intervention to traditional psychosocial intervention.	Efficacy of VR-based interventions could be compared with traditional psychosocial interventions for improving cognitive, social, and vocational skills as well as auditory verbal hallucinations or paranoia.
No studies included a VR control condition.	Perform RCTs that compare a VR experimental intervention to a VR control condition.	Isolate the impact of the intervention in VR vs the effects of using VR recreationally.
Inconsistency in the number and duration of VR-based sessions across studies.	Including various numbers and durations of VR-based sessions.	Determine the optimal number and duration of VR sessions to impact rehabilitation outcomes.
Sample sizes of the included studies were relatively small.	Use larger sample sizes.	More accurate results on the impact of VR-based interventions for individuals with psychosis.
Limited number of studies featuring follow-up assessments of the skills gained during the VR intervention.	Perform follow-up assessments.	Ensure that skills gained during the VR-based interventions are maintained and transferred into the real world.

^a^RCT: randomized controlled trial.

### Conclusions

This systematic review provided preliminary evidence that VR-based interventions may represent a novel and efficacious approach to improving psychosocial functioning in psychosis. VR-based psychosocial interventions were found to be safe and feasible. The VR-based interventions included in this review were shown to improve cognitive, social, and vocational skills as well as symptoms of auditory verbal hallucinations and paranoia in individuals with psychosis. The psychosocial skills learned from these interventions were also durable, with evidence supporting the maintenance and transfer of learned skills to real-world environments. Taken together, these findings reveal the potential of VR-based interventions to improve the persistent and debilitating symptoms of psychosis, which may be resistant to current pharmacotherapy. VR-based psychosocial interventions represent a promising adjunctive therapy for the treatment of psychosis, which may be used to improve psychosocial skills, community functioning, and quality of life in individuals with psychosis.

## Appendix

Multimedia Appendix 1 Summary of the risk of bias assessment scores of the studies included in this systematic review.

Multimedia Appendix 2 Detailed summary of the studies included in this systematic review.

## Abbreviations

RCT: randomized controlled trial
ToM: theory of mind
VR: virtual reality
