# Supplementary material for: Feasibility and Efficacy of Virtual Reality Interventions to Improve Psychosocial Functioning in Psychosis: Systematic Review
Source: JMIR Ment Health. 2022 Feb 18;9(2):e28502. doi: 10.2196/28502 (PMC8900915; doi:10.2196/28502)
Supplement: Multimedia Appendix 1 [file mental_v9i2e28502_app1.docx]

**Multimedia Appendix 1: Summary of the Risk of Bias Assessment Scores of the Studies Included in this Systematic Review**

**Supplemental Methods**

***Risk of Bias Assessment***

The risk of bias assessment of the RCTs was based on Version 2 of the Cochrane risk-of-bias tool for randomized trials (RoB 2) [40]. The excel tool to implement RoB 2 was used to evaluate the risk of bias of the RCTs based on the following domains:

1. randomization process
2. deviations from intended interventions
3. missing outcome data
4. measurement of the outcome
5. selection of the reported results

The overall risk of bias (identified as ‘Low’, ‘High’, or ‘Some Concerns’) for each domain was guided by the excel tool’s algorithm which used information from the evaluator’s assessment of signaling questions (each signaling question could be answered with ‘Yes’, ‘Probably Yes’, ‘Probably No’, ‘No’, or ‘No Information’). The overall risk of bias (identified as ‘Low’, ‘High’ or ‘Some Concerns’) for each RCT was guided by the excel tool’s algorithm based on the overall risk of bias for each domain. To assess the risk of bias of the randomized partial cross-over trial a modified version of the RoB 2 was used. This tool implemented the same principles as above, but it also included criteria relevant to the randomized partial cross-over trial such as bias due to a possible ‘carry-over’ treatment effect.

The risk of bias assessment of the non-randomized controlled trials were based on the Newcastle-Ottawa Quality Assessment Scale [43]. This tool evaluates non-randomized controlled trials based on the following domains:

1. selection
2. comparability
3. outcome

Each domain has a number of multiple-choice questions that are used to examine the quality of the study. ‘High’ quality choices are identified with a ‘star’ (a single question may have multiple ‘High’ quality choices). The selection domain examines four items and has a maximum of four ‘stars’. The comparability domain examines one item and has a maximum of two ‘stars’. The outcome domain examines three items and has a maximum of three ‘stars’. The quality of the non-randomized controlled trials was therefore evaluated out of a maximum of nine ‘stars’.

The risk of bias assessment of the single arm studies was based on a modified version of RoB 2 [42]. The selected signaling questions from the RoB 2 evaluated the following domains:

1. sampling
2. measurement
3. additional bias

Each signaling question was identified as ‘Low’, ‘High’, or ‘Some Concerns’. The overall risk of bias (identified as ‘Low’, ‘High’, or ‘Some Concerns’) was determined based on the answers to the signaling questions.

The risk of bias of each study was evaluated independently by two authors. Disagreements were resolved through discussion.

**Table S1.** Summary of the Risk of Bias Assessment Scores of the Randomized Controlled Trials

| **Study** | **Randomization Process** | **Deviations from the Intended Interventions** | **Missing Outcome Data** | **Measurement of the Outcomes** | **Selections of the Reported Result** | **Overall Bias** |
| --- | --- | --- | --- | --- | --- | --- |
| Chan et al. (2010) | Low | Low | Low | High | Low | Some Concerns |
| Delazizzo et al. (2021) | Some Concerns | *Effect of Assignment:*  Some Concerns  *Adherence to Assignment:* High | High | Low | Low | High |
| Geraets et al. (2020) | Low | Some Concerns | Low | Low | Low | Some Concerns |
| Park et al. (2011) | Low | Low | Some Concerns | High | Low | Some Concerns |
| Pot-Kolder et al. (2018) | Low | Some Concerns | Low | Low | Some Concerns | Some Concerns |
| Pot-Kolder et al. (2020) | Low | Some Concerns | Low | Low | Some Concerns | Some Concerns |
| Smith et al. (2015) | Some Concerns | Low | Low | Low | Low | Low |
| Tsang & Man (2013) | Some Concerns | Low | Low | Low | Low | Low |
| Vass et al. (2020) | Some Concerns | Low | Low | Low | Some Concerns | Some Concerns |

**Table S2.** Summary of the Risk of Bias Assessment Scores of the Non-randomized Controlled Trials

| **Quality assessment criteria** | **Acceptable (*)** | **La Paglia et al. (2013)** | **La Paglia et al. (2016)** |
| --- | --- | --- | --- |
| ***Selection*** | | | |
| Representativeness of exposed cohort? | Somewhat representative of the average ______________ in the community | ***** | ***** |
| Selection of the non-exposed cohort | Drawn from the same community as the exposed cohort | ***** | ***** |
| Ascertainment of exposure | Structured interview | ***** | ***** |
| Demonstration that outcome of interest was not present at the start of study? | Yes | ***** | ***** |
| ***Comparability*** | | | |
| Comparability of cohorts on the basis of the design or analysis | Study controls for any additional factor | ***** | ***** |
| ***Outcome*** | | | |
| Assessment of outcome? | No description | - | - |
| Was follow-up long enough for outcomes to occur? | Yes (select an adequate follow up period for outcome of interest) | **-** | ***** |
| Adequacy of follow up cohorts? | Complete follow up - all subjects accounted for | **-** | ***** |
| **Overall Quality Score (Maximum = 9)** | | **5** | **7** |

**Table S3.** Summary of the Risk of Bias Assessment Scores of the Single Arm Studies

| **Study** | **Sampling: Were the subjects in the study representative of the entire population from which they were recruited?** | **Measurement: Incomplete outcome data (attrition bias): Attrition bias due to amount, nature or handling of incomplete outcome data** | **Measurement: Do the analyses adjust for different lengths of follow-up of patients?** | **Measurement: Selective Reporting (reporting bias): Reporting bias due to selective outcome reporting** | **Additional Bias: Bias due to problems not covered elsewhere in the table** | **Overall Risk of Bias** |
| --- | --- | --- | --- | --- | --- | --- |
| Adery et al. (2018) | Some Concerns | Low | Low | Low | Low | Low |
| Amado et al. (2016) | Some Concerns | Low | Low | Low | Low | Low |
| Delazzizo et al. (2020) | Some Concerns | Low | Low | Low | High | High |
| Mortiz et al. (2013) | Some Concerns | Low | Low | Low | Low | Low |
| Rus-Calafell et al. (2014) | Some Concerns | Low | Low | Low | Low | Low |
| Sohn et al. (2016) | Some Concerns | Low | Low | Low | Low | Low |

**Table S4.** Summary of the Risk of Bias Assessment Scores of the Randomized Partial Cross-over Trial

| **Study** | **Randomization Process** | **Deviations from the Intended Interventions** | **Missing Outcome Data** | **Measurement of the Outcomes** | **Selections of the Reported Result** | **Overall Bias** |
| --- | --- | --- | --- | --- | --- | --- |
| du Sert et al. (2018) | Low | High | High | Low | High | High |
