# Supplementary material for: Feasibility and Efficacy of Virtual Reality Interventions to Improve Psychosocial Functioning in Psychosis: Systematic Review
Source: JMIR Ment Health. 2022 Feb 18;9(2):e28502. doi: 10.2196/28502 (PMC8900915; doi:10.2196/28502)
Supplement: Multimedia Appendix 2 [file mental_v9i2e28502_app2.docx]

**Multimedia Appendix 2: Detailed Summary of the Studies Included in this Systematic Review**

**Table S1.** Further Details of the Studies Included in this Systematic Review

| **Study** | **Age** | **Duration of VR Intervention(s)** | **VR System** | **Other Therapeutic Techniques Used in the Experimental Group** | **Feasibility** | **Measured Outcomes** |
| --- | --- | --- | --- | --- | --- | --- |
| Adery et al. (2018) [48] | *M* = 48.6  *SD* = 7.0 | VR Group:  Ten MASI-VR sessions (sessions were not timed, participants completed a set number of tasks during each session). The program occurred over approximately 5 weeks. | MASI-VR, a VR social skills training ‘game’ was developed by the researchers. This program was designed to model aspects of a social interaction. MASI-VR (non-immersive) was administered on a desktop as a video game. | N/A | The majority of participants reported that the training was extremely satisfactory (81.2%).  Participant retention rates were very high (89%). | - overall clinical symptoms - negative symptoms - positive symptoms - social engagement and function - participant retention rates - MASI-VR training acceptability |
| Amado et al. (2016) [44] | *M* = 38.6  *SD* = 12.1 | All participants:  Weekly 1-hour group VR sessions followed by a 20-minute group discussion. The intervention lasted for 3 months. | The virtual environments were developed and created by the  Laboratory of Memory and Cognition, LMC – Paris Descartes University, using the LMC software EditoMem (based on3DVIA Virtools Dev 5.0 software [3dVIA Virtools]). Participants used a joystick to navigate the virtual town and the Simulamem simulation software was used. | N/A | At week 12:  All participants reported a good tolerance, 3/8 participants asked for more intervention, especially a cognitive remediation program or a group for social cognition.  Two participants dropped out after the first two VR sessions. One participant refused to be evaluated after the program. | - clinical symptomatology - symptomatology and functional level - social autonomy - quality of life - self-esteem - insight dimension - attention - visual scanning abilities - processing speed - verbal and visual working memory - verbal learning - executive functioning - visuo-spatial abilities - retrospective memory - prospective memory - participant reports of functional gains and opinions on the intervention |
| Chan et al. (2010) [45] | All Participants:  Age >60    Experimental: *M* = 66.4  *SD* = 6.2  Control:  *M* = 65.9  *SD* = 5.5 | VR Group:  Ten VR sessions (2 activities, each 15 minutes long). Sessions occurred twice a week for 5 weeks. | The commercially available VR system, Interactive Rehabilitation Exercise System that builds in the Gesture Xtreme VR system was adapted for the VR program used in this study. Participants interact with the VR environment on a large screen using video gesture control technology. | N/A | No problems of simulator sickness.  The VR program improved volition to engage in VR activities.  100% retention rate. | - cognitive function (orientation, attention, comprehension, repetition, naming, constructions, memory, calculation, similarities, judgement) - simulator sickness - participants’ motivation within a natural or therapeutic environment |
| Dellazizzo et al. (2020) [55] | *M* = 43.4  *SD* = 14.6 | VR Group:  Nine weekly sessions consisting of 1 avatar  creation session and 8 therapeutic sessions where patients were  immersed into the VR setting. Of all participants, most (67%) received nine  sessions, whereas the rest received 7 sessions | Samsung GearVR and head-mounted display with a Samsung Galaxy S6 smartphone. The immersive environment consisted of an avatar standing in a dark seen from a first-person perspective. | The immersive therapeutic sessions consisted of pre-immersion  where the therapist would discuss the preceding week and determine the objective of the therapy session with the patients. During the intervention, an inventory of facial  expressions was integrated into the platform to use at the therapist’s  discretion to enable the avatar to express emotions that patients would easily recognize, such as joy, sadness, anger, and fear based on the Facial Action Coding System. | Patients noted that the VR intervention helped to embody their voice (i.e., auditory verbal hallucination) and make their experience come to life by enabling a direct discussion with their voice. When looking back into both therapies, patients expressed their complementarity and found the sequence to be the best option. | - auditory hallucinations - beliefs about voices - depression - symptomatology - quality of life, enjoyment, and satisfaction |
| Dellazizzo et al. (2021) [56] | *M* = 43.6  *SD* = 12.0 | VR Group:  Nine weekly sessions consisting of 1 avatar  creation session and 8 therapeutic sessions where participants were  immersed into the VR setting. Of all participants, most (67%) received 9  sessions, whereas the rest received 7 sessions. | Samsung GearVR and head-mounted display with a Samsung Galaxy S6 smartphone. The immersive environment consisted of an avatar standing in the dark seen from a first-person perspective. | The immersive therapeutic sessions consisted of pre-immersion  where the therapist would discuss the preceding week and determine the objective of the therapy session with the patients. During the intervention, an inventory of facial  expressions was integrated into the platform to use at the therapist’s  discretion to enable the avatar to express emotions that patients would easily recognize, such as joy, sadness, anger, and fear based on the Facial Action Coding System. | The VR intervention was found to be feasible to implement and acceptable to participants with no reported adverse events. | - auditory hallucinations - beliefs about voices - depression - symptomatology - quality of life, enjoyment, and satisfaction |
| du Sert et al. (2018) [57] | Range: 24-62  *M* = 42.9  *SD* = 12.4 | VR Group:  Seven weekly therapy sessions; 1 avatar creation session (the avatar was selected to most closely match that of the “persecutor”) and 6 45-minute therapeutic sessions. | Samsung GearVR and head-mounted display with a Samsung Galaxy S6 smartphone. The immersive environment consisted of an avatar standing in a dark seen from a first-person perspective. | The therapist reflected with the participant about the intervention and gathered information on what represents the hallucinatory experience. | Four participants did dropped out (from an original sample of 19) due to anxiety and lack of engagement in the treatment model. Furthermore, no participants were re-hospitalized during the study, but one participant did enter a counselling and support centre. | - psychiatric symptoms - auditory verbal hallucinations - life satisfaction - after visit 1, participants rated how much they felt in presence with their persecutor (0-10 scale) - after each VR-assisted therapy visit, participant rated their level of fear and anxiety (0-10 scale) |
| Geraets et al. (2020) [58] | Experimental: *M* = 38.1  *SD* = 10.0  Control:  *M* = 40.9  *SD* = 10.0 | Maximum: Sixteen 1-hour individual therapy sessions.  Minimum: Three sessions.  The 1-hour sessions included 40 minutes of VR exercises and 20 minutes of planning and reflecting on the exercises. Approximately 67% of the therapy time was spent in VR. | The Sony HMZ-T1/T2/T3 Head Mounted Display with a high-definition resolution of 1280×720 per eye, with 51.6 diagonal field of view, and a 3DOF tracker for head rotation. Participants used the Logitech F310 Gamepad to move within the environment. | Participants planned and reflected on the exercises with the psychologist. | The original sample consisted of 116 participants and 91 participants were available for analyses due to participants only being included in the analyses if baseline and posttreatment experience sampling method data were available and participants completed at least 3 sessions. Approximately 21.6% of the original sample was excluded. | - mental state (paranoia, negative affect, positive affect) |
| La Paglia et al. (2013) [46] | *M* = 33.0  *SD* = 12.3 | VR Group:  Ten weekly individual sessions lasting 90 minutes each. | NeuroVr2.0 software. | N/A | It was concluded that 90-minute sessions of VR do not allow the participant to fatigue or bore. | - Mini Mental State Examination - Frontal Assessment Battery - Trail Making Test - Tower of London - Memory Battery - Wisconsin Card Sorting Test - Stroop Colour World Test |
| La Paglia et al. (2016) [47] | Experimental:  *M* = 29.0  *SD* = 12.1  Control:  *M* = 35.0  *SD* = 9.9 | VR Group:  Ten VR sessions (90-minutes each). The program lasted for 10 weeks. | The VR environment was developed by researchers via the Neuro Vr2.0 software and the tasks were designed to train attention. Participants used head mounted displays, trackers, a computer, and a joypad to access the virtual environment. | N/A | No direct data on feasibility were reported. | - general cognitive functioning - executive function - sustained and divided attention - planning - brief and long-term memory - cognitive flexibility |
| Moritz et al. (2013) [59] | *M* = 40.5  *SD* = 9.9 | Participants were asked to walk a virtual street three times (1 practice trial; 2 experimental trials). | Unity3D, a game development editor with an integrated rendering engine. The artificial characters were created using the 3D modeling software DAZ-Studio. Body animations were based on “Biovision hierarchical” data from the motion capturing procedure. | N/A | No information on feasibility. | - paranoia symptomatology - accuracy - metamemory - affect recognition judgments |
| Park et al. (2011) [49] | Range:  18-45  Experimental:  *M* = 28.1  *SD* = 7.7  Control:  *M* = 31.2  *SD* = 7.7 | VR Group:  Ten group VR sessions (4-5 group members). The sessions were 90 minutes each. The program lasted for 5 weeks. | The methodological procedures were developed based on the guides by Bellack et al. (2004; Social Skills Training for Schizophrenia, Second Edition: A Step-by-Step Guide). | N/A | No simulator sickness reported.  Interest in the intervention and generalization of skills were both higher in the VR group.  There was no difference between groups in drop-out rate, but the VR group had a higher attendance rate. | - social skills (voice, nonverbal, and conversational skills) - assertiveness - interpersonal relationship skills - cognitive, affective, or behavioral responses to real life problem-solving situations - motivation - generalization - contribution of four social skills training constituents (material, content, therapist, and structure) to overall skill improvement - positive and negative symptoms - general psychopathology |
| Pot-Kolder et al. (2018) [60] | Range: 18-65  Experimental: *M* = 36.5  *SD* = 9.8  Control:  *M* = 39.5  *SD* = 10.0 | Sixteen 1-hour individual biweekly therapy sessions. The sessions consisted of 40 minutes of VR exercises and 20 minutes of planning and reflecting on the exercises. Approximately 67% of the therapy time was spent in VR. | The Sony HMZ-T1/T2/T3 Head Mounted Display with a high-definition resolution of 1280×720 per eye, with 51.6 diagonal field of view, and a 3DOF tracker for head rotation. Participants used the Logitech F310 Gamepad to move within the environment. | Participants planned and reflected on the exercises with the psychologist. | The average (and standard deviation) simulator sickness questionnaire total score was 57.5 (38.3) and 52.9 (41.9) after the fourth and eighth VR sessions, respectively. One participant dropped out due to nausea. Participants felt sufficiently present in the virtual environments. Eleven participants (19%) in the VR group dropped out of therapy (including 4 who never started treatment and 7 who discontinued treatment). Notably, 1 participant was too afraid and 2 found the head-mounted display too uncomfortable to tolerate. | - time spent with others - momentary paranoia - perceived social threat - momentary anxiety - ideas of persecution - ideas of social reference - safety behaviors - social interaction anxiety - depression inventory - quality of life - social functioning - stigma - jumping to conclusions – belief inflexibility - attention for threat - external attribution - social cognitive problems - subjective cognitive problems - negative self-core schema - negative others core schema - positive self-core schema - positive others core schema |
| Pot-Kolder et al. (2020) [61] | *See above description in Pot-Kolder et al. (2018).* | *See above.* | *See above.* | *See above.* | *See above.* | - cost-effectiveness ratio - cost (healthcare costs, travel costs, productivity costs, total societal costs) - paranoia - time spent with others - momentary anxiety - momentary paranoia - quality-adjusted life years |
| Rus-Calafell et al. (2014) [50] | Range:  18-55  *M* = 36.5  *SD* = 6.0 | VR Group:  Sixteen 1-hour sessions (30 minutes: discuss the social skills training intervention content; 30 minutes: use the VR technology) The program lasted for 8 weeks. | The *Soskitrain* VR program was developed by the researchers. Participants used a laptop with stereoscopic view, 3D glasses, and headphones to access the VR environment. | N/A | Participants reported a high level of satisfaction with the intervention’s benefits, the psychologist’s work, and acceptance of the VR system.  Fifteen patients were enrolled and 12 completed the study. | - positive and negative symptoms and general psychopathology - social performance and anxiety - social functioning - emotion perception - assertive behaviors - time spent in a conversation - participant satisfaction |
| Smith et al. (2015) [52] | Range: 18-55  Experimental: *M* = 40.8  *SD* = 12.2  Control:  *M* = 39.1  *SD* = 10.6 | VR Group:  Five VR sessions (up to 10 hours in total) over 5-10 business days. | The VR intervention was developed by a commercial company, SIMmersion LLC. The VR intervention is computer-based. | N/A | Participants reported that the VR intervention was enjoyable, easy-to-use, helpful, and prepared them for interviews. There was a VR session attendance rate of 90.0% and no participants dropped out during training. | - clinical symptoms - global cognitive ability - basic social cognition - advanced social cognition - VR acceptability - VR efficacy |
| Sohn et al. (2016) [53] | Range: 20-55  *M* = 36.7  *SD* = 5.4 | VR Group:  Eight weekly VR sessions (35 minutes each). | Convenience store and supermarket scenarios were selected because these are environments in which patients with schizophrenia are commonly employed. A commercial company, FNI Co., Ltd. (Gyeonggi Province, Korea) assisted the researchers with the development of the intervention.  Participants used a mouse to interact with the virtual environment which was presented on three liquid crystal display digital projectors. | N/A | One participant declined to participate in the study after being recruited. | - clinical condition - executive function - visual memory - verbal memory |
| Tsang & Man (2013) [54] | Range: 18-55  Experimental: *M* = 39.6  *SD* = 8.0  TAG:  *M* = 40.8  *SD* = 9.2  CG:  *M* = 41.6  *SD* = 9.9 | VR Group:  Ten VR sessions (30 minutes each). The program lasted for 5 weeks. | The VR program was developed by the researchers. It used a salesperson role because this is a common job for individuals with mental illness and it requires multiple social and problem-solving skills. Participants used a desktop computer, joystick, keyboard, mouse, monitor, and a set of stereo speakers to access the VR environment. | N/A | During the first session, one participant reported simulator sickness, but it was not a problem  throughout the rest of the training.  Participants reported that the VR-based training was more interesting and useful than conventional training.  Some participants (particularly those who were less educated and chronically ill with long-term deinstitutionalization and who rarely used computers) experienced different degrees of phobia.  From the initial group of 95 participants, 14 discontinued the intervention and 6 were lost to follow-up. | - global cognitive functioning - attention - memory - executive functioning - cognitive functioning at work - participants’ performance in sales-related activities - participants’ self-perceived ability in performing sales-related activities |
| Vass et al. (2020) [51] | Experimental:  *M* = 38.6  *SD* = 13.5  Control:  *M* = 48.8  *SD* = 8.9 | VR Group:  Nine weekly individual sessions (50 minutes each). The first session was designed to help participants understand the intervention/ technology. Then, there were 8 virtual simulation-based sessions. | Samsung's Gear VR equipment was used, including a head-mounted display, a Samsung S7 smartphone, and a Samsung Simple Controller. | Experiences of the simulation were discussed with a trained psychotherapist right after each task. Here, the therapist used cognitive and metacognitive techniques to help the patient in the recognition of consistencies. | Patients found this novel intervention interesting, engaging, easy, and safe to use. | - Baron-Cohen Mind in the Eyes Test - Positive and Negative Syndrome Scale - Wisconsin Card Sorting Test - Repeated Battery for the Assessment of Neuropsychological Status - Cartoon Test - Metaphor-irony Test - faux pas overall - attribution of intention - recognize false belief - empathy - Lancashire Quality of Life Profile |

CG: conventional group; *M*: mean; MASI-VR: Multimodal Adaptive Social Intervention in Virtual Reality; *SD*: standard deviation; TAG: therapist-administered group; VR: virtual reality.
